# IJBNPA in 2016: Strategy for advancing the science of behavior change in nutrition and physical activity, and associated editorial priorities

**DOI:** 10.1186/s12966-016-0404-8

**Published:** 2016-07-11

**Authors:** Russell Jago, Lesley Wood

**Affiliations:** Centre for Exercise, Nutrition & Health Sciences, School for Policy Studies, University of Bristol, 8 Priory Road, Bristol, BS8 1TZ UK

## Abstract

The goal of the International Journal of Behavioral Nutrition and Physical Activity (IJBNPA) is to be the leading diet and physical activity journal. To achieve this aim we embrace and publish a number of different research designs from small, but in depth, qualitative studies to large scale cohort studies. IJBNPA prioritises research based on randomised controlled trials (RCTs), systematic reviews (with or without meta-analyses, as appropriate), and well conducted observational studies that expand knowledge and understanding of the area. IJBNPA will also consider and publish other study designs that are of sufficient quality such as strong or ground-breaking methodological papers, rigorous qualitative studies, debate papers and commentaries. However, due to the demands on the journal, we publish pilot studies only in exceptional circumstances and we do not publish protocol papers or letters to the editors. The goal of this editorial is to highlight to our readers and authors the process by which we identify which papers to review and publish along with our editorial priorities.

## Criteria for assessment

The first issue of IJBNPA was published in 2004, since when the number of publications has increased steadily to its current level of around 160 articles per year. However, the number of submissions has also risen dramatically and we are expecting in excess of 700 submissions for 2016. The rise in the number of submissions and the quality of the papers is a testament to work of authors, reviewers and editors. It does, however, mean that editorial decisions about which papers to send for peer review, which to accept, and which to reject are becoming increasingly more challenging. Journal editing and reviewing is a “volunteer activity” and there is a limit to the number of papers for which we can find high quality reviewers. As an editing team we are therefore faced with daily decisions about which papers should be published and we use two fundamental criteria to inform our decisions: novelty and methodological rigour and a paper has to meet both criteria to be considered for peer review. In terms of novelty, editors are asked to consider if the paper represents a new contribution to the evidence. While we view replication of findings to be an essential component of research, there has to be an upper limit on the number of times a study is replicated. After several studies have been conducted on the same topic it is more likely that a systematic review, rather than another small empirical study, will advance understanding. We therefore ask authors to justify clearly in their cover letter the uniqueness of their study and its key contribution to evidence.

Methodological rigour is essential for ensuring that results are reproducible and answer the question posed [1]. Thus, when assessing papers that have been submitted to IJBNPA, and particularly over the past 18 months, we have placed an increasing emphasis on the rigour of the methods used to collect and analyse data. Along with other changes, we have revised some of the submission requirements. It is now a journal requirement that all randomised controlled trials are prospectively registered to be eligible for peer review. Any RCT that is uploaded without this information will be automatically rejected either by the system or by the Managing Editor. In addition, we require all authors of RCTs to complete a CONSORT checklist [2] and flowchart, and to use the TIDIER framework [3] to describe intervention components. TIDIER is particularly important for a behavioural journal such as IJBNPA as it encourages authors to describe all intervention components, giving transparency to the study and allowing the diet and physical activity community to fully understand all elements of the intervention and the constituent components. This understanding is vital for assessing the future usefulness and application of different interventions. For observational studies we ask that all authors complete the STROBE checklist [4] with the recently released STROBE-nut required for nutrition papers [5]. For systematic review and meta-analyses authors are required to complete the PRISMA checklist [6, 7]. For all empirical studies we want to know how the sample was recruited, how representative the sample was of the target group, how the analysed sample differed from the recruited sample and how any missing data were handled. In all instances, these checklists and additional materials should be uploaded as an additional file at the point of submission. For qualitative studies we ask that authors are transparent about the processes by which participants were recruited, how they assessed the trustworthiness of their data, how debriefing occurred, as well as providing full details of the analytic process/method (including any triangulation).

## Setting the agenda

A key goal of the editing team is for IJBNPA to set the agenda for diet and physical activity research. To achieve this we hope to become a home for both empirical papers and thought provoking debate papers. A debate paper provides the opportunity for authors to present conflicting views [8] or synthesise evidence on an important diet and physical activity topic [9]. As such, this type of paper can play a pivotal role in setting the research agenda and we encourage our colleagues to consider submitting papers in this category. The motto of our parent society, the International Society of Behaviour Nutrition and Physical Activity (ISBNPA), is “Advancing Behaviour Change Science” and in line with this a key goal of IJBNPA is to publish ground-breaking methodological papers that support the field in meeting this aspiration. We are receptive to papers that outline new statistical techniques, reinforcements of existing statistical techniques [10], the application of health economics to diet and physical activity research [11], the development of new scales and instruments [12] and any other methodological advance that enables the field to develop.

## Summary

The goal of the IJBNPA editorial team is to publish the highest quality studies to advance the science of behaviour change in relation to diet, physical activity and related behaviours. As part of this goal we will publish papers in a variety of formats, including debate papers, which have the potential to initiate discussion and challenge the current orthodoxy.
